# Determinants of early chronic kidney disease in patients with recently diagnosed type 2 diabetes mellitus: a retrospective study from the Taiwan Diabetes Registry

**DOI:** 10.1186/s12882-024-03567-1

**Published:** 2024-04-15

**Authors:** Yun-Kai Yeh, Kuan-Hung Lin, Wayne Huey-Herng Sheu, Su-Huey Lo, Yen-Po Yeh, Chien-Ning Huang, Chii-Min Hwu, Chieh-Hsiang Lu

**Affiliations:** 1 Section of Endocrinology and Metabolism, Department of Medicine, Taipei Veterans General Hospital, No. 201, Sec. 2, Shipai Rd., Beitou District, Taipei City, 112 Taiwan R.O.C.; 2 Department of Medicine, National Yang Ming Chiao Tung University Hospital, No. 169, Xiaoshe Rd., Yilan County, 260 Taiwan R.O.C.; 3 Institute of Molecular and Genomic Medicine, National Health Research Institutes, No. 35, Keyan Road, Zhunan Town, Miaoli County, 350 Taiwan R.O.C.; 4 Faculty of Medicine, National Yang Ming Chiao Tung University School of Medicine, No. 155, Sec. 2, Li-Nong Street, Beitou District, Taipei, 112 Taiwan R.O.C.; 5 Tao-Yuan General Hospital, Ministry of Health and Welfare, No. 1492, Zhongshan Rd., Taoyuan Dist, Taoyuan City, 330 Taiwan R.O.C.; 6 Changhua County Public Health Bureau, No. 162, Sec. 2, Jhongshan Rd., Changhua County, 500 Taiwan R.O.C.; 7 Institute of Medicine, Chung Shan Medical University Hospital, No. 110, Section 1, Jianguo North Road, Taichung City, 402 Taiwan R.O.C.; 8 Section of Endocrinology and Metabolism, Department of Internal Medicine, Ditmanson Medical Foundation, Chiayi Christian Hospital, No. 539 Jhongsiao Rd., Chia-Yi City, 600 Taiwan R.O.C.; 9 Lutheran Medical Foundation, Kaohsiung Christian Hospital, No. 86, Huasin St., Lingya Dis., Ksohsiung City, 802 Taiwan

**Keywords:** Type 2 diabetes, Early chronic kidney disease, Taiwan diabetes registry

## Abstract

**Background:**

We tried to identify the risk factor associate with early chronic kidney disease (CKD) in recently diagnosed type 2 diabetes mellitus patients by utilizing real-world data from Taiwan Diabetes Registry.

**Materials and methods:**

Patients with type 2 diabetes mellitus recently diagnosed within 1 year. We divided the study participants into control group and early CKD group. Early CKD was defined as either CKD stage G1 with albuminuria, CKD stage G2 with albuminuria, or CKD stage G3a regardless of albuminuria (Urine-albumin to creatinine ratio (UACR) ≥ 3 mg/mmol). Control group was defined as CKD G1 or CKD G2 without albuminuria. Logistic regression analyses were used to compare differences in clinical characteristics between the subgroups. Linear regression models were employed to examine the factors predicting estimated glomerular filtration rate (eGFR) and UACR.

**Results:**

Total 2217 patients with recently diagnosed type 2 diabetes mellitus were included. 1545 patients were assigned to control group and 618 patients were assigned to the early CKD group. Age (odds ratio (OR) 1.215, 95% confidence interval [CI] 1.122–1.316), systolic blood pressure (OR 1.203, 95% CI 1.117–1.296), glycated hemoglobin (OR 1.074, 95% CI 1.023–1.129) and triglyceride (OR 2.18, 95% CI 1.485–3.199) were found to be significant risk factors. Further, presence of bidirectional association between UACR and eGFR was found.

**Conclusions:**

We reported factors associated with early CKD in recently diagnosed type 2 diabetes mellitus patients. Variables that associated with eGFR and UACR were identified respectively, included a mutual influence between UACR and eGFR.

**Supplementary Information:**

The online version contains supplementary material available at 10.1186/s12882-024-03567-1.

## Background

The global prevalence of type 2 diabetes mellitus, a common metabolic disorder, has been increasing rapidly in recent years [1]. As a result, the comprehensive care and prevention of diabetic complications, including microvascular and macrovascular complications, have become a major challenge that requires more attention. Asymptomatic dysglycemia can persist for months to years before the diagnosis of type 2 diabetes mellitus is established. During this period, even low-grade hyperglycemia can contribute to vascular damage and cause microvascular or macrovascular events. By the time of diagnosis, patients may have already developed some diabetic complications. Previous studies have shown that diabetic kidney disease (DKD) has the highest prevalence among diabetic complications when type 2 diabetes mellitus is diagnosed [2, 3]. DKD is also a leading cause of diabetes-related cardiovascular disease and mortality [4], resulting in high medical costs and healthcare burdens. Therefore, DKD is an crucial complication that needs to be considered in the care of type 2 diabetes mellitus.

A study conducted by the Chronic Kidney Disease Prognosis Consortium found that the hazard ratios for end-stage renal disease (ESRD) at different estimated glomerular filtration rate (eGFR) levels of 60, 45, and 15 ml/min per 1.73 m2 were 4, 29, and 454, respectively, compared with an eGFR of 95 ml/min per 1.73 m2. In the same study, hazard ratios for ESRD at urine albumin-to-creatinine ratios (UACR) of 30, 300, and 1000 mg/g were 5, 13, and 28, respectively, compared with a UACR of 5 mg/g [5]. Other studies have also shown that lower eGFR and higher albuminuria are associated with higher adverse events [6, 7]. The Kidney Disease: Improving Global Outcomes (KDIGO) has categorized chronic kidney disease (CKD) into different stages using eGFR and UACR [8]. In patients with type 2 diabetes mellitus and early CKD, studies have demonstrated that multifactorial intervention has long-term benefits for complications and mortality [9, 10]. Therefore, the detection and management of early stage CKD in patients with type 2 diabetes mellitus have been proposed and emphasized [8, 11].

DKD is also the major cause of ESRD in Taiwanese patients who require regular hemodialysis or peritoneal dialysis [12]. It is essential to identify the population with diabetes who are at risk of developing DKD. Previous literature has indicated that increased age, body mass index (BMI), levels of cholesterol, triglycerides, and glycated hemoglobin are associated with an increased likelihood of developing diabetic kidney disease in recently diagnosed patients with type 2 diabetes mellitus. On the contrary, no history of diabetic retinopathy, high levels of high-density lipoprotein cholesterol, and non-smoking status are associated with a reduced risk [13, 14]. However, the above studies primarily focused on recently diagnosed patients with type 2 diabetes mellitus, and limited information is available regarding the determinants of CKD, particularly in the early stage, among individuals with recently diagnosed type 2 diabetes mellitus. Our study aims to identify the factors associated with early CKD in patients with type 2 diabetes mellitus diagnosed within one year in Taiwan. Furthermore, we seek to explore the relative contributions of modifiable clinical factors for eGFR and UACR in the study cohort.

## Materials and methods

### Study subject and ethic

The Taiwan Diabetes Registry (TDR) is a national, observational, and prospective registry conducted by the Diabetes Association of the Republic of China (R.O.C., Taiwan). The primary objective of the registry is to understand the status of diabetes care and risk factors for complications in patients with diabetes. The TDR program involves the collaboration of 14 medical centers, 44 regional and local hospitals, and 37 general practice clinics. The TDR includes participants with both Type 1 diabetes mellitus (T1DM) and type 2 diabetes mellitus, with informed consents obtained from all patients. Clinical information and diabetes-related medical records are collected using an electronic portal web-based platform. This study has been approved by the Joint Institute Review Board in Taiwan (protocol number: 14-S-012).

For this study, we selected type 2 diabetes mellitus patients from TDR who were diagnosed within one year and enrolled between October 1, 2015 and August 31, 2018. This was a cross-sectional study. Only the data recorded on the first registration was utilized. Patients without available eGFR were excluded from the study. Patients without available urinary UACR was also excluded.

### Study design

We divided the study participants into a control group and an early CKD group in accordance with the KDIGO 2012 stratification [8]. The control group consisted of patients without CKD, defined as either CKD stage G1 without albuminuria or stage G2 without albuminuria. Modification of Diet in Renal Disease (MDRD) equation was used to calculate eGFR [15], expressed in ml/min/1.73 m2. Albuminuria was defined as a UACR$\ge$ 3 mg/mmol. In contrast, the early CKD group comprised patients with early-stage CKD, defined as either CKD stage G1 with albuminuria, CKD stage G2 with albuminuria, or CKD stage G3a regardless of albuminuria. We analyzed data and variables between the two groups to identify risk factors for early-stage CKD in patients with type 2 diabetes mellitus.

### Data collection

We collected information on the general characteristics of the study participants, such as gender, age, body mass index (BMI), diabetes duration, blood pressure, and waist circumference. In addition, we collected laboratory data, including HbA1c, fasting glucose level, lipid profile, renal function, liver function, and UACR.

### Statistical analysis

Continuous variables were analyzed by using t-tests to evaluate mean values and standard deviations. We used the Chi-square test to determine if there were significant differences between two categorical variables. Additionally, we conducted univariate and multivariate analyses by using logistic regression models to compare the differences in clinical characteristics between the subgroups (with or without early CKD), while considering confounding variables. Furthermore, linear regression models were employed to examine the factors predicting estimated eGFR and UACR in the recently diagnosed type 2 diabetes mellitus patients, regardless of the independence among variables. All statistical analyses were conducted by SPSS software (version 23.0). A *p*-value < 0.05 was considered statistically significant.

## Results

**Fig. 1:**
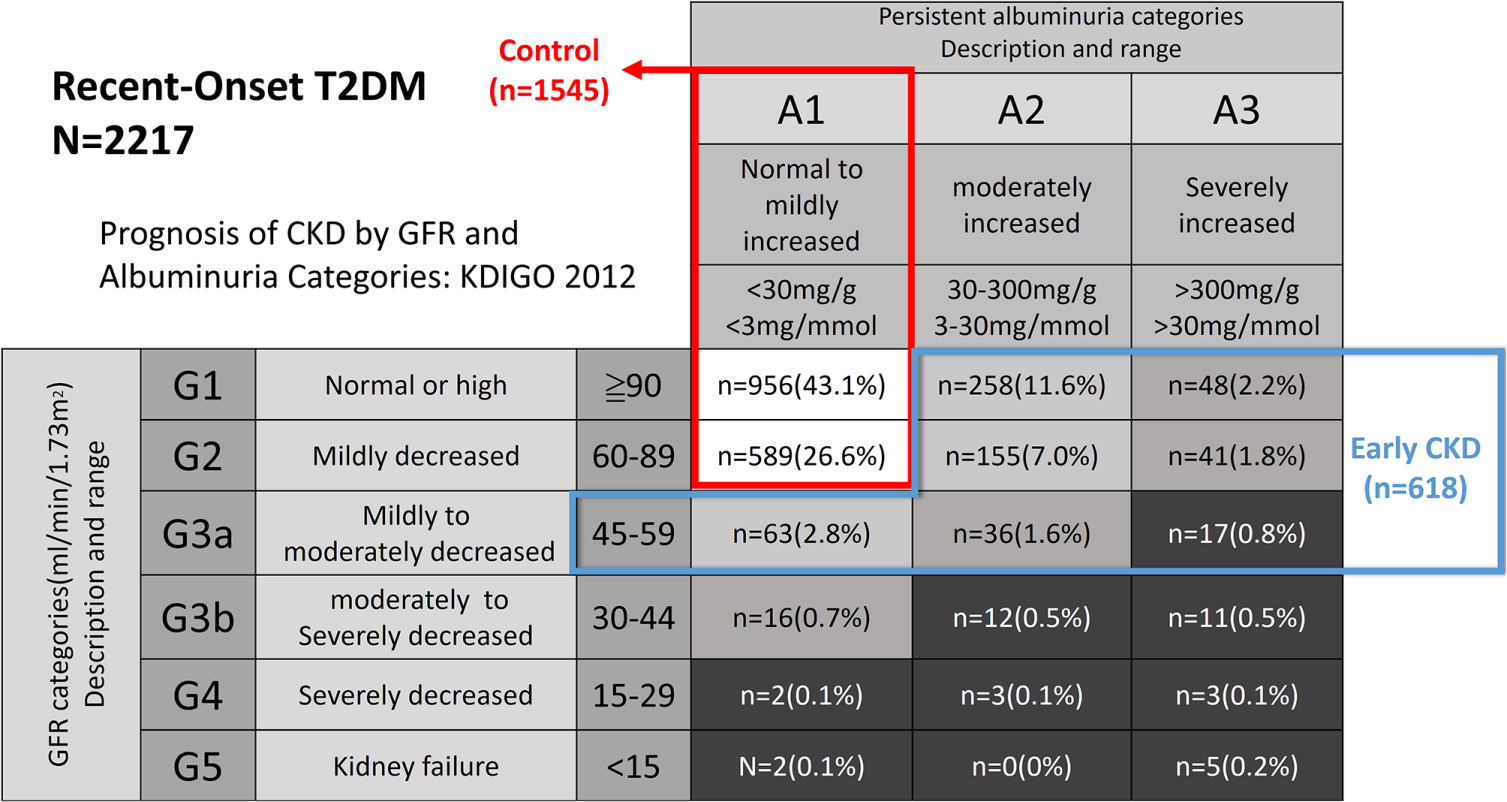
Distribution of patients who were enrolled in different chronic kidney disease (CKD) categories based on Kidney Disease Improving Global Outcomes (KDIGO) 2012. Control group was comprised of patients with CKD stage G1 without albuminuria or stage G2 without albuminuria. Early CKD group was comprised of patients with CKD stage G1 with albuminuria, CKD stage G2 with albuminuria, or CKD stage G3a regardless of albuminuria

From October 1, 2015, to August 31, 2018, a total of 2217 patients with type 2 diabetes mellitus diagnosed within one year and available eGFR with UACR data were enrolled in TDR. Of these patients, 1545 patients (69.7%) did not have CKD and were assigned to the control group. The remaining 672 patients (30.3%) had CKD, with 618 (27.9%) meeting the early CKD definition mentioned above and being classified into the early CKD group (Fig. 1). Baseline characteristics of the control and early CKD groups are shown in Table1 1. Compared to the control group, the early CKD group had a significantly lower eGFR and a significantly higher log UACR. Additionally, the early CKD group had significantly higher age, systolic blood pressure (SBP), diastolic blood pressure (DBP), fasting blood glucose, HbA1c, creatinine, and triglyceride levels. However, there was no significant difference in sex, diabetes duration, body weight, waist circumference, BMI, total cholesterol, alanine aminotransferase (ALT), and low-density lipoprotein cholesterol (LDL-C) between the two groups (Table 1).

**Table 1:** Descriptive characteristics of the study participants † Continuous variables are expressed as mean (standard deviation, SD)

	Control (*n* = 1545)	Early CKD (*n* = 618)	*p*
Sex (M/F)	865/680	358/260	0.677
Age (years)	54.2 (13.3)	57.1 (13.6)	< 0.001*
Diabetes duration (years)	0.08 (0.58)	0.09 (0.63)	0.681
BW (kg)	71.9 (16.0)	71.9 (15.1)	0.913
WC (cm)	91.0 (11.2)	92.07 (10.9)	0.051
BMI (kg/m^2^)	27.1(9.0)	27.4 (8.5)	0.407
Systolic BP (mmHg)	128.7 (16.0)	134.6 (18.4)	< 0.001*
Diastolic BP (mmHg)	77.5 (11.0)	79.58 (12.4)	< 0.001*
Fasting glucose (mmol/L)	8.26 (3.51)	9.18 (4.12)	< 0.001*
HbA_1c_ (%)	8.131 (2.287)	9.094 (8.156)	0.004*
TC (mmol/L)	4.79 (1.14)	4.90 (1.27)	0.073
TG (mmol/L)	1.81 (1.90)	2.21 (2.37)	-
Log TG	0.17 (0.26)	0.24 (0.28)	< 0.001*
LDL-C (mmol/L)	2.84 (0.90)	2.84 (1.00)	0.873
ALT (ukat/L)	0.58 (0.45)	0.59 (0.45)	0.686
Cr (umol/L)	70.7 (15.9)	79.6 (23.9)	< 0.001*
eGFR (mL/min/1.73 m^2^)	100.89 (28.19)	93.16 (53.39)	0.001*
UACR (mg/mmol)	1.10 (0.81)	31.36 (80.64)	-
Log UACR	-0.09 (0.41)	1.01 (0.60)	< 0.0001*

CKD, chronic kidney disease; BW, body weight; WC, waist circumference; BMI, body mass index; BP, blood pressure; HbA_1c_, glycated hemoglobin; TC, total cholesterol; TG, total triglyceride; Log, log transformation; LDL-C, low-density lipoprotein cholesterol; ALT, alanine aminotransferase; Cr, creatinine; eGFR, estimated glomerular filtration rate; UACR, urine albumin/creatinine ratio

Data are express ed as mean ± S.D. Two-sample *t* tests or χ [2] tests are used to compare the differences between the two groups

Table 2 presents the results regarding the differences in clinical characteristics between two subgroups of recently diagnosed type 2 diabetes mellitus patients: those with early CKD and those without. The analysis revealed significant associations between age, SBP, HbA1c and triglyceride levels with the presence of early CKD in recently diagnosed type 2 diabetes mellitus patients (Table2 2). Higher Age, SBP, HbA1c and triglyceride levels were more likely to develop early CKD in recently diagnosed type 2 diabetes mellitus patients.

**Table 2:** Univariate and multivariate logistic regression model of modifiable clinical variables associated with early CKD in patients with recently diagnosed type 2 diabetes

	Univariate analysis	*P value*	Multivariate analysis	*P value*
	Odds ratio (95% confident interval)		Odds ratio (95% confident interval)	
BW (per 10 kg)	0.997 (0.939, 1.058)	0.913	-	-
WC (per 10 cm) Age(per 10 years)	1.086 (0.999, 1.181) 1.177 (1.096, 1.265)	0.052 < 0.001	- 1.215 (1.122, 1.316)	- < 0.001*
Systolic BP (per 10 mmHg)	1.229 (1.162, 1.300)	< 0.001	1.203 (1.117, 1.296)	< 0.001*
Diastolic BP (per 10 mmHg)	1.172 (1.080, 1.271)	< 0.001	0.984 (0.881, 1.099)	0.775
Fasting glucose (mmol/L)	1.064 (1.039, 1.090)	< 0.001	1.026 (0.993, 1.059)	0.122
HbA_1c_ (%)	1.098 (1.058, 1.139)	< 0.001	1.074 (1.023, 1.129)	0.004*
TC ( mmol/L)	1.078 (0.996, 1.166)	0.061	-	
Log TG	2.656 (1.868, 3.777)	< 0.001	2.180 (1.485, 3.199)	< 0.001*
LDL-C ( mmol/L)	1.009 (0.912, 1.115)	0.866	-	

CKD, chronic kidney disease; BW, body weight; WC, waist circumference; BMI, body mass index; BP, blood pressure; HbA_1c_, glycated hemoglobin; TC, total cholesterol; TG, total triglyceride; Log, log transformation; LDL-C, low-density lipoprotein cholesterol; ALT, alanine aminotransferase; Cr, creatinine; eGFR, estimated glomerular filtration rate; UACR, urine albumin/creatinine ratio

The results of the analysis examining clinical variables predicting eGFR in recently diagnosed type 2 diabetes mellitus are shown in Table 3. The analyses revealed that body weight, SBP, fasting glucose, and UACR were significantly associated with eGFR. In particular, SBP and UACR were identified as negative predictors for eGFR. These findings indicated that higher levels of SBP and UACR were associated with lower eGFR in these patients (Table 3).

**Table 3:** Univariate and multivariate linear regression analyses of modifiable clinical variables in predicting estimated glomerular filtration rate in patients with recently diagnosed type 2 diabetes

	Univariate analysis	*P* value	Multivariate analysis	*P* value
	β (95% confident interval)		β (95% confident interval)	
BW (per 10 kg)	1.720 (0.704, 2.736)	0.001	1.549 (0.498, 2.601)	0.004*
WC (per 10 cm)	-0.008 (-1.447, 1.431)	0.991	-	
Systolic BP (per 10 mmHg)	-1.527 (-2.464, -0.591)	0.001	-1.423 (-2.395, -0.451)	0.004*
Diastolic BP (per 10 mmHg)	1.154 (-0.233, 2.541)	0.103	-	
Fasting glucose (mmol/L)	1.054 (0.623, 1.485)	< 0.001	0.921 (0.449, 1.393)	< 0.001*
HbA_1c_ (%)	0.562 (0.226, 0.898)	0.001	0.340 (-0.014, 0.694)	0.060
TC (mmol/L)	0.911 (-0.440, 2.262)	0.186	-	
Log TG	8.175 (2.120, 14.229)	0.008	5.703 (-0.735, 12.141)	0.082
LDL-C (mmol/L)	-0.504 (-2.201, 1.193)	0.561	-	
Log UACR	-5.501 (-7.762, -3.240)	< 0.001	-5.531 (-7.859, -3.203)	< 0.001*
			*R*^*2*^ = 0.033	< 0.001*

CKD, chronic kidney disease; BW, body weight; WC, waist circumference; BMI, body mass index; BP, blood pressure; HbA_1c_, glycated hemoglobin; TC, total cholesterol; TG, total triglyceride; Log, log transformation; LDL-C, low-density lipoprotein cholesterol; ALT, alanine aminotransferase; Cr, creatinine; eGFR, estimated glomerular filtration rate; UACR, urine albumin/creatinine ratio

Table 4 presents the results of the analysis examining the clinical variables predicting UACR in recently diagnosed type 2 diabetes mellitus. The study found that SBP, fasting blood glucose, log triglyceride level, and eGFR were significantly associated with UACR. Of note, higher levels of SBP, fasting blood glucose, and log triglyceride were positively associated with UACR, while eGFR showed a negative association. The analyses also indicated that UACR and eGFR were mutually influenced by each other, which suggests the presence of bidirectional associations between these two variables (Table 4).

**Table 4:** Univariate and multivariate linear regression analyses of modifiable clinical variables in predicting urine albumin/creatinine ratio ‡ in patients with recently diagnosed type 2 diabetes

	Univariate analysis	*P* value	Multivariate analysis	*P* value
	β (95% confident interval)		β (95% confident interval)	
BW (per 10 kg)	-0.010 (-0.029, 0.009)	0.312	-	
WC (per 10 cm)	0.022 (-0.005, 0.049)	0.103	-	
Systolic BP (per 10 mmHg)	0.085 (0.067, 0.102)	< 0.001	0.082 (0.061, 0.104)	< 0.001*
Diastolic BP (per 10 mmHg)	0.070 (0.045, 0.096)	< 0.001	-0.014 (-0.046, 0.018)	0.393
Fasting glucose (mmol/L)	0.021 (0.013, 0.029)	< 0.001	0.018 (0.009, 0.027)	< 0.001*
HbA_1c_ (%)	0.007 (0.001, 0.014)	0.019	0.003 (-0.004, 0.009)	0.379
TC (mmol/L)	0.030 (0.005, 0.055)	0.018	-0.013 (-0.040, 0.014)	0.356
Log TG	0.305 (0.194, 0.417)	< 0.001	0.241 (0.116, 0.366)	< 0.001*
LDL-C (mmol/L)	0.015 (-0.016, 0.0047)	0.340	-	
eGFR (mL/min/1.73 m [2])	-0.002 (-0.003, -0.001)	< 0.001	-0.002 (-0.003, -0.001)	< 0.001*
			*R*^*2*^ = 0.069	< 0.001*

CKD, chronic kidney disease; BW, body weight; WC, waist circumference; BMI, body mass index; BP, blood pressure; HbA_1c_, glycated hemoglobin; TC, total cholesterol; TG, total triglyceride; Log, log transformation; LDL-C, low-density lipoprotein cholesterol; ALT, alanine aminotransferase; Cr, creatinine; eGFR, estimated glomerular filtration rate; UACR, urine albumin/creatinine ratio

‡ after log transformation

## Discussion

In this study, we observed that approximately 30.3% of patients diagnosed with recently diagnosed type 2 diabetes mellitus also exhibited CKD, with the majority (92%) being in the early stages of the disease. Age, SBP, HbA1c and triglyceride levels were associated with the presence of early CKD in recently diagnosed type 2 diabetes mellitus patients. We also identified several clinical variables, including body weight, SBP, fasting glucose, and UACR, that exhibited significant associations with eGFR. Furthermore, we found that SBP, fasting blood glucose, log triglyceride level, and eGFR were significantly associated with UACR in this cohort. Notably, our findings also indicated a mutual influence between eGFR and UACR.

The association between type 2 diabetes mellitus and DKD has been extensively discussed in numerous studies. Various factors have been identified to be associated with CKD. In a German study, a comparison was made between type 2 diabetes mellitus patients with or without CKD using large databases [16]. The study specifically focused on type 2 diabetes mellitus patients with an eGFR less than 60 mL/min/1.73 m2 or those with an eGFR greater than 60 mL/min/1.73 m2 but with albuminuria (UACR > 30 mg/g). The study’s findings revealed that type 2 diabetes mellitus patients with CKD were older, had a longer duration of type 2 diabetes mellitus, had a higher incidence of higher triglyceride levels, and were more likely to be female. Furthermore, a significantly higher level of HbA1c and blood pressure were observed in type 2 diabetes mellitus patients with CKD. These results were similar to our study, except that we did not find a higher percentage of female patients in the CKD group, and our study only included patients recently diagnosed with type 2 diabetes mellitus. Another cross-sectional study also identified old age and an HbA1c level greater than 7% as significant risk factors for the development of advanced CKD (eGFR < 60 mL/min/1.73 m2) in patients with type 2 diabetes mellitus [17]. In contrast to our study, we specifically focused on recently diagnosed type 2 diabetes mellitus patients with early-stage CKD. Regardless of the stage of CKD, old age and higher HbA1c levels were both significant risk factors for the development of CKD in type 2 diabetes mellitus patients.

Consistent with prior literature, SBP exhibited a negative correlation with eGFR [18, 19]. Additionally we found that fasting blood glucose was positively associated with eGFR, which is in line with previous study [20]. The positive association indicates higher fasting blood glucose may reflect kidney hyperfiltration, even though there’s no significant association between eGFR and HbA1c in our study. We additionally conducted a subgroup analysis focusing on patients with CKD stage G3a. The results revealed no significant association between fasting blood glucose and eGFR under univariate linear regression (Table S1). There’s study demonstrated the same trend that HbA1c had positive association with eGFR in patients with early CKD. However, when the disease progresses to CKD stage 3 or 4, the association between HbA1c and eGFR becomes negative [21]. Proper glycemic control remains crucial in slowing the decline of eGFR and improving renal function. Our findings regarding the association between body weight and eGFR differ from the previous study that reposted an association between higher BMI and worsened eGFR [22]. However, it is worth noting that conflicting results exist in the literature. Another study conducted by the Hong Kong Diabetes Registry revealed a negative correlation between CKD and BMI [23]. One hypothesis suggests that CKD may lead to malnutrition and sarcopenia, which could contribute to lower BMI. Due to the conflicting results, the role of body weight or BMI in predicting CKD in patients with recently diagnosed type 2 diabetes mellitus remains uncertain. Further research is requiring to better understand the impact of body weight on CKD in this population.

In this study, our findings are consistent with previous studies that have shown a positive association between SBP and fasting glucose levels with UACR [18, 19, 24]. Interestingly, we found that triglyceride levels were also positively associated with UACR, which has also been reported in previous research [25]. The underlying pathophysiological mechanism for this association is still unclear. However, it has been suggested that elevated triglyceride levels may impact the glomerulus and lead to glomerular injury.

This study also demonstrated that eGFR and UACR mutually influenced each other. Previous studies have shown that lower albuminuria is associated with CKD regression [26], and reduced eGFR may be associated with albuminuria [27]. Additionally, other studies have reported that albuminuria and declining eGFR are both predictors of worsened cardiovascular or renal outcomes [28, 29]. Just like a vicious cycle, albuminuria can lead to declining eGFR, and reduced eGFR can also result in more albuminuria. Therefore, early intervention to prevent the worsening of eGFR and albuminuria is important in patients with DKD.

This study focused on individuals with early CKD, specifically those in KDIGO CKD stage $\le$G3a. Previous research has shown that all-cause mortality increases from 1.1 per 100 person-years in CKD stage G3a to 4.8 per 100 person-years in CKD stage G3b [30]. Hence, identifying the early CKD population is crucial, and early intervention may improve outcomes.

Patients with DKD are at an increased risk of other diabetic complications, such as hypoglycemia, ketoacidosis, retinopathy, neuropathy, and cardiovascular events [8]. Given the potential impact of DKD on patient health, multidisciplinary and comprehensive care should be offered to patients with DKD to prevent further progression of the disease. Lifestyle modifications such as smoking cessation and avoiding nephrotoxic agents should be emphasized, along with secondary prevention of cardiovascular events through the use of aspirin and statins. Sodium-glucose cotransporter-2 inhibitors and glucagon-like peptide-1 receptor agonists have emerged as the drugs of choice for patients with DKD due to their proven benefits beyond glycemic control. In addition, angiotensin-converting enzyme inhibitors /angiotensin II receptor blockers should be used as first-line therapy for blood pressure control in DKD patients, unless contraindicated [8, 31].

The strength of this study lies in its use of a nationwide, multicenter diabetes registry cohort and the analysis of a large number of cases. Furthermore, the study focused on a group with early CKD, an area that has been sparsely investigated. The study also highlighted the possible risk factors for developing early CKD in type 2 diabetes mellitus diagnosed within one year and demonstrated the mutual influence between eGFR and albuminuria. However, there are also some limitations to the study. First, as it is a cross-sectional analysis, the causal relationship between the variables cannot be ascertained. Further research with longitudinal designs and broader patient populations is warranted to better understand the dynamic relationship between type 2 diabetes mellitus, CKD, eGFR, and UACR. Second, the inclusion of patients with type 2 diabetes mellitus diagnosed within one year may limit the generalizability to those with longer disease duration. Third, as the data was obtained from a single time-point in a registry, we cannot ascertain whether the low eGFR values were chronic or transient. Fourth, our study did not analyze lifestyle factors, which may also play a role in the development of early CKD. Finally, the R-squared value of the linear regression was small in this study. We believe that there may be additional factors beyond type 2 diabetes mellitus that could influence eGFR and UACR in the development of early CKD, which we were unable to identify in the current database. Further research in this regard could be considered. However, despite the small R-squared value, the *p*-values for the linear regressions are significant, indicating an association between the variables, eGFR, and UACR.

In conclusion, this study provides valuable insights of CKD in patients with type 2 diabetes mellitus diagnosed within one years, particularly in the early stages. The results of our study suggest that several factors are associated with an increased risk of developing early CKD in recently diagnosed patients with type 2 diabetes mellitus. Specifically, older age, higher levels of HbA1c, and elevated triglyceride levels were found to be significant risk factors. Besides, body weight, SBP, and fasting blood glucose are modifiable clinical variables that are closely associated with eGFR. Additionally, other factors such as SBP, fasting blood glucose, and triglyceride levels are associated with UACR. Furthermore, the findings indicate a mutual influence between UACR and eGFR. The study underscores the importance of managing modifiable factors in slowing the progression of CKD in patients with recently diagnosed type 2 diabetes mellitus. Further research is needed to evaluate the effects of such management strategies.

### Electronic supplementary material

Below is the link to the electronic supplementary material.

Supplementary Material 1

## Data Availability

The datasets used and analyzed during the current study are available from the corresponding author on reasonable request.
