# Supplementary material for: Determinants of early chronic kidney disease in patients with recently diagnosed type 2 diabetes mellitus: a retrospective study from the Taiwan Diabetes Registry
Source: BMC Nephrol. 2024 Apr 15;25:133. doi: 10.1186/s12882-024-03567-1 (PMC11017602; doi:10.1186/s12882-024-03567-1)
Supplement: Supplementary file 1 — Supplementary Material 1 [file 12882_2024_3567_MOESM1_ESM.docx]

**Table S1** Univariate linear regression analyses of modifiable clinical variables in predicting estimated glomerular filtration (eGFR) rate in patients with KDIGO stage G3a (45≦eGFR<60 mL/min/1.73 m^2^ ) (n=116)

|  | Univariate analysis | |
| --- | --- | --- |
|  | β (95% confident interval) | *P value* |
| Fasting plasma glucose  HbA1c | -0.027 (-0.266, 0.212)  -0.128 (-0.267, 0.011) | 0.825  0.072 |

HbA_1c_, glycated hemoglobin
